# Physical Therapists in Primary Care Are Interested in High Quality Evidence Regarding Efficacy of Therapeutic Ultrasound for Knee Osteoarthritis: A Provincial Survey

**DOI:** 10.1155/2013/348014

**Published:** 2013-06-04

**Authors:** Norma J. MacIntyre, Jason W. Busse, Mohit Bhandari

**Affiliations:** ^1^School of Rehabilitation Science, McMaster University, IAHS 403, 1400 Main Street W, Hamilton, ON, Canada L8S 1C7; ^2^Departments of Anesthesia and Clinical Epidemiology & Biostatistics, McMaster University, Health Sciences Centre, Room 2V9, 1280 Main Street W, Hamilton, ON, Canada L8S 4K1; ^3^Department of Surgery, McMaster University, 293 Wellington Street N, Suite 110, Hamilton, ON, Canada L8L 8E7

## Abstract

Recent high-level evidence favours therapeutic ultrasound (US) for reducing pain in people with knee osteoarthritis (OA). It is unknown how current practice patterns align with current evidence regarding US efficacy and whether physical therapists perceive a need for further high-level evidence. We conducted a descriptive electronic survey to characterize the beliefs and use of US among physical therapists in Ontario treating people with nonsurgical knee OA. Most of the 123 respondents (81%) reported at least some use of US with 45% using it often or sometimes. The main goal for using US was to reduce pain in the surrounding soft tissue (*n* = 66) and/or the knee joint (*n* = 43). Almost half (46%) endorsed the belief that US is likely to be beneficial for clients with nonsurgical knee OA. Most respondents (85%) expressed interest in the results of a randomized controlled trial evaluating the effectiveness of US on pain and physical function. Patterns of use reflect the respondents' belief that US is likely to be beneficial for knee OA pain.

## 1. Introduction

Osteoarthritis (OA) is the most common type of arthritis and knee OA, being highly prevalent, accounts for as much or more lower extremity disability in North American community-dwelling older adults than any other disease [1]. No disease-modifying treatment exists, and knee OA may progress from a dynamic process of injury and repair to irreversible joint damage requiring joint replacement to treat the unrelenting pain and/or significant disability [2]. As the population ages and at the same time is becoming increasingly heavier, the prevalence of knee OA and the associated economic and personal burden are expected to rise [2]. Nonsurgical management is an important first step to prevent disability and maintain quality of life in the growing number of people with knee OA.

Current clinical practice guidelines (CPGs) for the management of nonsurgical knee OA recommend using a combination of pharmacologic and nonpharmacologic interventions—a number of which are offered by physical therapists. In clinical practice, physical therapists tailor multicomponent interventions to the needs of the individual with knee OA in order to attain the goals of treatment. For example, physical agents may be administered as adjunctive to exercise interventions. Efficacy of therapeutic ultrasound (US) is of particular interest as this is the physical agent most commonly used by physical therapists for treatment of painful musculoskeletal conditions and, therefore, widely available [3, 4]. Despite the fact that US is an adjunctive therapy and the effectiveness of specific combinations of interventions for knee OA has yet to be established, three recent rigorous syntheses of the best available evidence suggest that US administered by physical therapists reduces pain and may improve physical function in this population [5–7]. However, recommendations in current CPGs are discordant. Three CPGs for management of nonsurgical knee OA recommend that US should not be used [8–10]; one guideline recommends use [11]; two guidelines provide no guidance for or against the use of US due to poor quality, contradictory evidence available for review [12, 13]; four guidelines do not include US among the treatment options considered because systematic reviews conducted to that point in time could not draw definitive conclusions [14–17]. 

Theoretical, biological, and clinical rationales for the use of US in the management of nonsurgical knee OA have been reported. Therapeutic acoustic radiation is transmitted into the target tissue via US as high-frequency pressure waves generated by a piezoelectric crystal in the sound head of the US device. These pressure waves produce mechanical effects and/or thermal effects aiming to heat the deeper tissues to increase blood flow, local metabolism, tissue regeneration, and collagen elasticity, decrease an inflammatory response and/or enhance soft tissue healing [3]. The nonthermal mechanical effects are proposed to be achieved through the application of pulsed, low intensity US [3]. *In vitro* studies using articular cartilage chondrocyte cell cultures demonstrate that low intensity US can induce chondrocyte proliferation and production of extracellular matrix [18–21]. A number of studies using animal models of cartilage injury to evaluate the effect of US on the rate of cartilage degeneration have shown benefits [22–26]. In some of these *in vitro* studies, pulsed low intensity US with temporal average intensities achievable using devices widely available in physical therapy practice has been used with beneficial effects on cartilage repair [22–24]. In other studies, very low intensity pulsed US such as that used in bone healing systems (temporal average intensity = 0.03 W/cm^2^) has been used [25, 26]. Very low intensity pulsed US slowed progression of cartilage degeneration in the guinea pig model of idiopathic OA—particularly in those guinea pigs with early rather than established degeneration [25]. The studies in the animal models of OA have shown that a therapeutic dose between 36 and 300 J/cm^2^ stimulates the mechanotransduction pathway and enhances cartilage formation, regeneration, and extracellular matrix formation. These observations suggest that very low intensity pulsed US could stimulate the repair of injured cartilage and, if applied at early stages, may slow the progression of knee OA. To our knowledge, only two clinical trials have explored the biologic effects of US on cartilage in people with knee OA [27, 28]. One RCT, reported as an abstract [27] with the data acquired and reviewed by our group [5], used an indirect measurement of knee cartilage injury and reported that arthritis severity was reduced by pulsed US (temporal average intensity of 0.625 W/cm^2^, 24 × 15 min sessions over 8 weeks) in knee OA participants who fell within the lower and middle tertile for a scintigraphy-based “arthritis severity index” at baseline. A pilot RCT suggested that pulsed US (temporal average intensity of 0.2 W/cm^2^, 24 × 9.5 min sessions over 8 weeks) may increase cartilage thickness in people with knee OA who attend >80% of treatments [28]. Whereas structural change is hypothesized to have an important impact on OA burden, a strong association between knee OA joint structural changes and clinical symptoms has not been reported. Nevertheless, two meta-analyses conducted subgroup analyses based on mode (pulsed US (*n* = 3 trials/177 patients and knees) and continuous US (*n* = 4 trials/221 patients and knees)) using two different approaches and found that the magnitude of improvement in knee OA pain was greater with pulsed US (temporal average intensity between 0.375 and 0.625 W/cm^2^ and a therapeutic dose <150 J/cm^2^) [5, 6].

Given the recent evidence favouring US for reducing knee OA pain, the potential for improving physical function and stimulating cartilage repair, and the equipoise in the CPGs, it is unclear whether physical therapists are currently using US for the management of knee OA and/or perceive an evidence gap. The purpose of our study was to describe beliefs and use of US among primary care physical therapists in Ontario treating people with nonsurgical knee. 

## 2. Materials and Methods

### 2.1. Ethics

The study protocol for our cross-sectional survey of members of the Ontario Physiotherapy Association (OPA) was approved by the McMaster University Faculty of Health Sciences/Hamilton Health Sciences Research Ethics Board. The first page of our survey presented the study information and explained that completion of the survey constituted consent to participate. The survey could be exited at any time; however, responses were anonymous so data for a specific individual could not be removed from the database after responses were submitted.

### 2.2. Questionnaire Development

 Previous literature [29, 30] and input from 4 academic physical therapists with expertise in OA informed the development of an English-language electronic questionnaire. We pretested the questionnaire with an independent group of 4 clinical physical therapists with expertise in OA. Following revisions to shorten the length, the survey was converted to electronic format for distribution and data collection using Qualtrics survey software (Qualtrics Labs, Inc., Provo, UT, USA).

Respondents were asked to complete 5 questions to determine demographics and confirm eligibility. Respondents indicated their gender (female/male), age (21 to 30 y, 31 to 40 y, 41 to 50 y and >50 y), years in practice (<5, 5 to 10, 10 to 20 and >20), and the average number of clients with nonsurgical knee OA the respondent personally treats in one year (1 to 20, 21 to 40, >40, and never treated clients with nonsurgical knee OA). Those who indicated that they never treat clients with nonsurgical knee OA were automatically exited from the survey. The 5 questions which asked about the use of US for the management of nonsurgical knee OA are shown in Table 1. An open textbox was placed after question 3 with the following instructions: “please explain the factors that influence your treatment decision regarding use of ultrasound.” Respondents were also asked to identify clinical outcome measures they used for clients with nonsurgical knee OA by selecting from a checklist of 17 response options naming outcome measures for assessing pain intensity, stiffness, generic quality of life, condition and region-specific self-reported physical function questionnaires, performance-based measures of physical function/mobility, arthritis self-efficacy, and/or “other” and specifying additional outcome measures in a textbox as appropriate. 

### 2.3. Questionnaire Administration

Approximately 60% of college registrants are members of the OPA, and these members could be contacted by electronic and postal mail addresses. An invitation to complete the electronic survey was sent to all members by e-mail June 23, 2011. An article in the OPA's June/July newsletter *Physiotherapy Today* served as an initial reminder, and monthly e-mail reminders were sent until the survey was closed on September 30, 2011. Individual anonymized responses were collected in an electronic database downloaded for analyses. 

### 2.4. Sample Size

A sample size of 100 was targeted to achieve a margin of error of 8% at the 95% confidence level based on the assumption that respondents would provide divergent answers that reflect the uncertainty in the evidence and the number of physical therapists licensed to practice in Ontario who were likely to treat people with knee OA in a primary care setting provided by the College of Physiotherapists of Ontario (*n* = 294). The sample size was determined using an online survey sample size calculator (http://www.surveysystem.com/sscalc.htm).

### 2.5. Data Analysis

The response rate for each question was calculated by dividing the number of respondents selecting a given response option by the total number of respondents. Open-ended responses were summarized by frequency of themes. 

## 3. Results

### 3.1. Characteristics of Respondents

The demographics of the 123 respondents are summarized in Table 2. Assuming that 100% of our target samples of 294 registrants have membership in the Ontario Physiotherapy Association (rather than the estimated 60%), the response rate may be as low as 42%.

### 3.2. Attitudes and Current Use of Ultrasound

**Figure 1:**
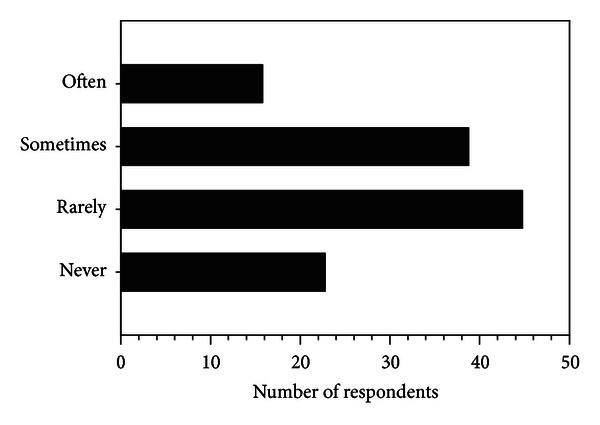
Response rates for options to complete the stem “For clients with nonsurgical knee osteoarthritis, I use ultrasound therapy.”

**Figure 2:**
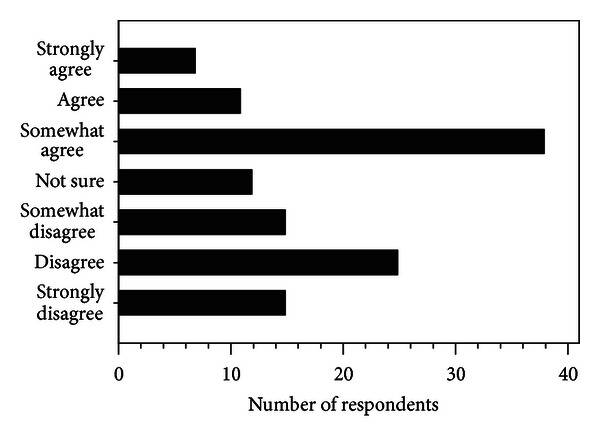
Response rates for options to complete the stem “For clients with nonsurgical knee osteoarthritis, ultrasound is likely to be beneficial.”

**Figure 3:**
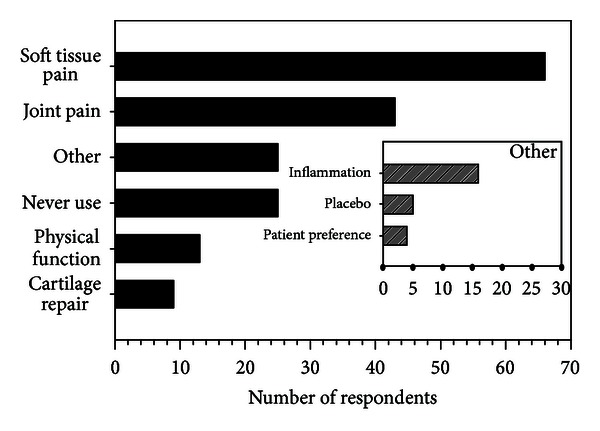
Response rates for options to complete the stem “I use ultrasound in clients with nonsurgical knee osteoarthritis to (mark all that apply).” Inset illustrates the reasons given by respondents selecting the response option “other.”

Figure 1 illustrates that the majority of respondents (81%; 100 of 123) reported using US to manage nonsurgical knee OA “rarely” to “often.” While 81% of respondents reported using US, only 56 respondents (46%) endorsed belief in US's efficacy as shown in Figure 2.  Figure 3 summarizes the reasons why respondents use US for knee OA with most aiming to reduce pain in the surrounding soft tissues and/or the knee joint. Factors which influenced the decision to use US included lack of quality or convincing evidence (60%), clinical experience/belief that time is better spent on treatment options that are less passive for the client and more time efficient for the physical therapist (27%), lack of access to the modality or insufficient visits (12%), and lack of biological plausibility for benefit (1%). 

### 3.3. Treatment Efficacy Required for Routine Use of US for Clients with Nonsurgical Knee OA

**Figure 4:**
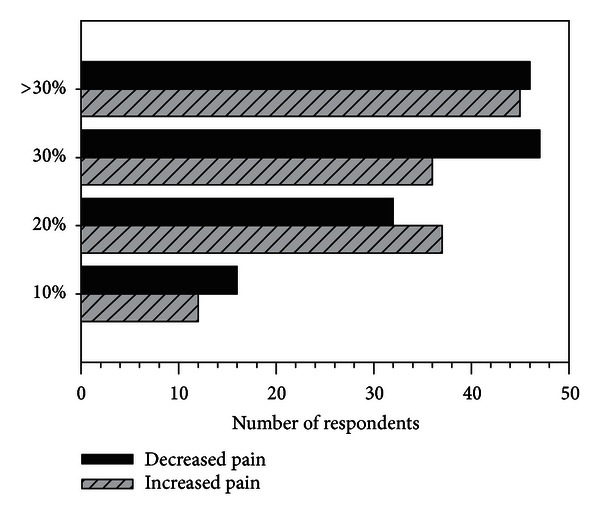
Response rates for options to complete the stem “I would use ultrasound in clients with nonsurgical knee osteoarthritis, if high quality evidence using ultrasound demonstrates the following improvements in my clients on average (mark all that apply).”

The magnitude of improvement, on average, which respondents set as the criteria for using US is depicted in Figure 4; the most frequently selected response options were 30% or greater reduction in pain and more than 30% increase in physical function. Eight respondents indicated that they would not use US regardless of the magnitude of the improvement. Most respondents expressed interest in the results of a randomized controlled trial evaluating the effectiveness of US on pain and physical function (40% “strongly agree,” 35% “agree,” and 10% “somewhat agree”). The remainder of the participants selected the response options “not sure” (2%), “disagree” (2%), and “strongly disagree” (11%). 

### 3.4. Clinical Outcome Measures Used for Clients with Nonsurgical Knee OA

Most reported using the numeric rating scale (NRS) for assessing pain intensity (80%) and the Lower Extremity Functional Scale for assessing physical function (68%). A number reported using the pain intensity VAS (39%) and the Timed Up and Go test (24%). Consistency in the use of other measures of impairment, self-reported physical function, balance and mobility, walking performance, arthritis self-efficacy, and quality of life dropped dramatically (≤15% for all).

## 4. Discussion

We surveyed physical therapists treating clients with nonsurgical knee OA in a direct access/primary care setting in Ontario. Attitudes and use of US among physical therapists treating these clients are divergent. Almost half (45%) use US “often” or “sometimes” while the majority (55%) use US “rarely” or “never” to treat knee OA pain. A similar proportion (54%) does not believe it is likely to be beneficial for clients with nonsurgical knee OA. However, some of the 23 respondents who report never using US identified health service barriers as reasons for this decision. In some service delivery models used in Ontario, the number of funded visits precludes effective implementation of US therapy. Moreover, there is economic disincentive to provide passive therapeutic modalities requiring longer treatment times for the physiotherapist given that the remuneration for each visit is unchanged by the length or number of treatment components. Thus factors other than the therapist's belief regarding efficacy influence the decision not to use US. 

Current CPGs for the management of nonsurgical knee OA reflect the limitations in the evidence available at the time of their development [8–17]. Recent meta-analyses report that US (10 to 24 sessions continuous or pulsed) is effective for reducing pain [5–7] and may be beneficial for improving physical function [5, 7]. Low intensity pulsed US (varying from 0.375 to 0.625 W/cm^2^  delivered over 10 to 24 sessions) appeared to produce greater benefit and eliminated the heterogeneity of the pooled data [5, 6]. However these results must be interpreted with caution because only 3 of the 6 trials available for synthesis used low intensity pulsed US [27, 31, 32], and these were all conducted by the same research group. A systematic review published in 2012 added one new trial to the meta-analysis (387 participants; pulsed or continuous US) and found statistical and clinical improvements in both pain and physical function [7]. Although these systematic reviews are encouraging, the small sample sizes and low methodological quality of the randomized controlled trials available for meta-analysis limit confidence in the conclusions. Consistent with these methodological limitations, most of our respondents expressed interest in the results of a high quality randomized controlled trial to determine the effects of US on knee OA.

Our respondents suggest that practice patterns will not change given proof of efficacy unless the magnitude of improvement at the level of the individual client is at least 30% for pain and greater than 30% for physical function on average. Expectations for a reduction in pain of 30% or greater are in agreement with published Minimal Clinically Important Difference (MCID) values [33]. For example, a pain intensity score of 6 on the 11-pt NRS would need to decrease by at least 1.8 points, and the MCID for this outcome measure is 2 points [33]. In keeping with this, Wang et al. [7] estimated that US changed pain scores on the VAS by −16.3 (95% CI: −20.9 to −11.7) cm. The criterion for improvement in physical function can be interpreted based on reported average scores of around 50 points on the Lower Extremity Functional Scale for people with nonsurgical knee OA receiving physical therapy interventions [28, 34]. An increase of at least 18 points would be required in order to meet the criterion for greater than 30% improvement. In contrast, the MCID is reported to be 9 points over a 6-month interval in this patient group [35]. However, Wang et al. [7] estimated that US changed WOMAC function scores by −21.2 (95% CI: −29.8 to −12.8) points. Thus the magnitudes of clinical improvement in pain and physical function estimated based on the US efficacy trials meta-analyzed by Wang et al. [7] are consistent with those desired by our respondents to support the decision to use US in the management of people with knee OA.

Our survey findings need to be interpreted in the context of the limitations. E-mail invitations were distributed to physical therapists who were members of the OPA, and we are uncertain how many of our target sample are hold membership in the OPA. We sampled physical therapists working in Ontario where primary health care services have been restructured within the context of the chronic care model in which the individual assumes greater responsibility for monitoring and managing the signs and symptoms of knee OA in collaboration with their direct access health care professionals [36]. In Ontario, as in many parts of the world, physical therapists are self-regulated, primary health care professionals who provide diagnosis and treatment of acute and chronic conditions within the scope of practice to the public without need of referral. In keeping with primary care reform, a number of respondents reported that their decision to use US was influenced by their belief that treatment time is better spent on active treatments and education in skills required for successful self-management during periods of functional stability. The factors that influence treatment decisions regarding use of US may differ among those working within other health care models. We did not prompt respondents to report constraints which influenced their use of US such as number of treatment sessions or access to the modality. These factors were only captured through textbox responses. Finally we did not ask respondents to report the mode and intensity of US used nor the combination of interventions they administer with (or without) US. Notwithstanding these limitations, our results do provide a starting point for understanding the current practice patterns and beliefs regarding the use of US for clients with nonsurgical knee OA.

## 5. Conclusion

The use of US for the management of clients with nonsurgical knee OA is variable; however more than 80% of physical therapists surveyed use US, at least on rare occasions, despite the fact that only 46% endorsed the belief that US was likely to benefit the client. The primary rationale for applying US is to treat soft tissue pain. Most physical therapists are interested in the results of a high quality randomized controlled trial to determine the effect of US on knee OA pain and physical function in people with nonsurgical knee OA.

## Figures and Tables

**Table 1:** Survey questions regarding attitudes and behaviors around therapeutic ultrasound.

Question stem	Response options
(1) For clients with nonsurgical knee OA, I use ultrasound therapy:	◯ Often ◯ Sometimes ◯ Rarely ◯ Never

(2) For clients with nonsurgical knee OA, ultrasound is likely to be beneficial:	◯ Strongly disagree ◯ Disagree ◯ Somewhat disagree ◯ Not sure ◯ Somewhat agree ◯ Agree ◯ Strongly agree

(3) I use ultrasound in clients with nonsurgical knee OA to: (mark all that apply)	◯ Reduce pain in surrounding soft tissue ◯ Reduce joint pain ◯ Improve physical function ◯ Stimulate cartilage repair ◯ Other (please specify) ◯ I do not use ultrasound in clients with nonsurgical knee OA

(4) I would use ultrasound in clients with nonsurgical knee OA if high quality evidence using ultrasound demonstrated the following improvements in my clients on average (mark all that apply):	◯ 10% reduction in pain ◯ 10% improvement in physical function ◯ 20% reduction in pain ◯ 20% improvement in physical function ◯ 30% reduction in pain ◯ 30% improvement in physical function ◯ >30% reduction in pain ◯ >30% improvement in physical function ◯ I would not use ultrasound regardless of the findings of high quality evidence

(5) I would be interested in the results of a randomized controlled trial evaluating the effectiveness of low intensity pulsed ultrasound on pain and physical function:	◯ Strongly disagree ◯ Disagree ◯ Somewhat disagree ◯ Not sure ◯ Somewhat agree ◯ Agree ◯ Strongly agree

**Table 2:** Participant demographics.

Characteristic	*N*
Gender	
Male/female	33/90
Age	
21 to 30 y	22
31 to 40 y	36
41 to 50 y	30
>50 y	35
Years in practice	
<5 y	19
5 to 10 y	21
11 to 20 y	34
>20 y	49
Average number of clients with nonsurgical knee osteoarthritis treated per year	
1 to 20	43
21 to 40	42
>40	38
